# Supplementary material for: Inhibitors of Mitochondrial Human Carbonic Anhydrases VA and VB as a Therapeutic Strategy against Paclitaxel-Induced Neuropathic Pain in Mice
Source: Int J Mol Sci. 2022 Jun 2;23(11):6229. doi: 10.3390/ijms23116229 (PMC9181376; doi:10.3390/ijms23116229)
Supplement: Supplementary file 1 [file ijms-23-06229-s001.zip › ijms-1715471-supplementary.pdf]

Supplementary Materials

Inhibitors of mitochondrial human carbonic anhydrases VA and VB as a therapeutic strategy against paclitaxel induced neuropathic pain in mice

Supplementary Table S1. Cell viability assay

	Cell viability %
***5d** concentration (µM)*	*24 h incubation*
0	*100 ± 2.3*
10	*98.4 ± 1.6*
30	*102.6 ± 1.8*
100	*94.6 ± 5.3*

*RBE4 cells (4 × 10^4 cell/well) were treated with increasing concentrations of **5b** (10 µM, 30 µM and 100 µM). Incubation was allowed for 24 h. Cell viability was measured by the MTT assay. The control condition was arbitrarily set as 100 % and values expressed as mean ± SEM of three experiments.*
